# Open Access to General Practice Was Associated with Burnout among General Practitioners

**DOI:** 10.1155/2013/383602

**Published:** 2013-01-20

**Authors:** Peter Vedsted, Ineta Sokolowski, Frede Olesen

**Affiliations:** The Research Unit for General Practice, Institute of Public Health, Aarhus University, Bartholins Alle 2, 8000 Aarhus C, Denmark

## Abstract

Walk-in open access in general practice may influence the general practitioner's (GP's) work, but very little research has been done on the consequences. In this study from Danish general practice, we compare the prevalence of burnout between GPs with a walk-in open access and those without. In a questionnaire study (2004), we approached all 458 active GPs in the county of Aarhus, Denmark, and 376 (82.8%) GPs returned the questionnaire. Walk-in open access was defined as at least 30 minutes every weekday where patients could attend practice without an appointment. Burnout was measured by the Maslach Burnout Inventory. Analyses using logistic regression were adjusted for gender, age, marital status, job satisfaction, minutes per consultation, practice organisation, working hours, number of listed patients per GP, number of contacts per GP, continuing medical education- (CME-) activities, and clusters of GPs. In all, 8% of GPs had open access and the prevalence of burnout was 24%. GPs with walk-in open access were more likely to suffer from burnout. Having open access was associated with a 3-fold increased likelihood of burnout (OR = 3.1 (95% CI: 1.1–8.8, *P* = 0.035)). Although the design cannot establish causality, it is recommended to closely monitor possible negative consequences of open access in general practice.

## 1. Background

Quick access to general practice is considered to be a major patient need compelling general practitioners (GPs), policy makers, and administrators to plan how access is managed. Management models promoting quick access have been known as open access, same-day access, walk-in, and advanced access and cover a variety of different aspects of access: patients can attend within a specified time period each day without an appointment, patients can access the GP's calendar to make their appointment themselves, and specific standards for waiting time. The aims of open access have primarily been to individualise the types of appointments available and thus increase service by making it possible for patients to get an appointment with their preferred GP thereby securing continuity, minimising unnecessary visits and waiting times, and optimising appointment scheduling to boost capacity, better match capacity to demand, and reduce patient no-shows [1–3].

The benefits of open access seem to be unclear and research has so far focused on waits and patient experiences. However, in England 20% of GPs using advanced access reported a lack of resources, and some expressed concerns about the trade-off between immediate access and continuity of care [4]. Further, a postal survey in the UK found that 40% of GPs with open access used appointments only available for booking on the same day [2]. Thus, open access may seem to have undesirable effects on the GPs' working conditions by adding to existing pressures on GPs in general [5, 6] and hence raising the risk of stress and burnout [7–9] that would hamper the quality of general practice care [10–12].

All GPs in Denmark are independent contractors with the public scheme (the regional health authorities) and they are fully responsible for the organisation of the work in their practice. In Denmark, some practices have had time slots with open access each day for years, allowing patients to attend without appointment. The consequences for the GPs have never been investigated, and the aim of this study was to investigate the association between being a GP in a practice with walk-in open access and experiencing burnout in a sample of Danish GPs.

## 2. Methods

Some 40% of GPs work in single-handed practices. GPs in partnership practices share the same patient list. GPs act as gate-keepers and 99% of citizens are registered with a particular general practice which they have to consult. List size is on average 1.550 patients per GP (including children). According to the national contract, the practice has to be open from 8 AM till 4 PM, from Monday to Friday, and, in addition, for two hours a week outside normal working hours. All GPs are available on phone for advice, usually every weekday from 8 AM to 9 AM. Acute patients should be seen the same day and nonacute patients within five weekdays. There is no contract on open access and virtually all GPs make use of an appointment scheme. However, by tradition, some practices provide walk-in consultations for acute conditions, typically provided every day during a 1-hour prebooked time slot. These time slots are much shorter, often 5 minutes, than usual consultations of 10–15 minutes in Denmark. Patients come without an appointment and can be seen for a specific medical problem and will need to get a new appointment if the problem cannot be solved in the open access consultation.

### 2.1. Study Population

In a cross-sectional postal questionnaire study, all 458 active GPs in the county of Aarhus, Denmark, by May 2004, were invited to participate. The 126 nonresponders were sent a reminder with a new questionnaire after four weeks. GPs were remunerated in the amount of 16 € for responding. Four GPs were excluded: one being a member of the research group and three were on leave. The questionnaires were returned to the research unit for optical scanning.

### 2.2. Questionnaire

The 10-page self-administered questionnaire included scales on burnout, job satisfaction, and questions about practice organisation, continuing medical education (CME) activities, and GP characteristics. Walk-in open access was defined as at least 30 minutes prebooked walk-in every weekday where patients could attend without an appointment. Practices with open access less often were not included in the group. This was done to reproduce a situation as close to complete open access as possible. 

Burnout was measured by the Maslach Burnout Inventory-Human Services Survey (MBI-HSS), which is an international scale that is considered valid [13–15] and which has been translated and adapted into Danish using standardised procedures (forward, backward, expert meetings, and pretest) and the psychometric performance can therefore be transferred. This self-rating-scale consists of 22 items forming three subscales. Each item is scored on a 7-point ordinal scale. The three subscales are (1) emotional exhaustion (9 items), (2) depersonalization (5 items), and (3) personal accomplishment (8 items). Each dimension receives a score which, relative to normative population scores, is categorized as low or high. The definition of burnout was a high score on the subscale emotional exhaustion (>26) and/or on depersonalization (>9) [13]. Because only a few GPs in open access practices had high burn out, we did not consider the score “high burn out” in this study. Job satisfaction was measured by the Job Satisfaction Scale by Warr, Cook, and Wall which consists of ten items scored on a 7-point scale [16].

### 2.3. Analysis

For each GP we calculated the scores for burnout. We calculated the association between suffering from burnout and being a GP in a practice with open access. The association was calculated as odds ratio (OR) in a logistic regression model. We calculated the crude OR and an adjusted OR including age, gender, marital status (married, single), job satisfaction, practice organisation (solo practice, group practice, or partnership practice), minutes typically used per consultation divided by the median (<13, >12), number of enrolled patients per GP (<1385, 1385–1785 and >1785 patients), weekly working hours (<37, 37–41, 42–46, 47–51, and >51 hours), and number of consultations provided per GP in 2003 (<7720, 7720–10770, >10770). We have earlier found an association between burnout and participation in CME [10]. Therefore, we included as confounding variables whether the GP was a member of a CME or a supervision group and data on the number of days during the past two-year period spent on conferences, courses, and seminars relevant to general practice (0–5, 6–9, 10–17, 18+ days).

As some of the GPs were working in the same practice, we corrected for clusters of GPs within the same practice using robust variance estimates. We calculated 95% confidence intervals (95% CI) for proportions and ratios. *P* values of 5% or less were considered statistically significant. Data was analysed using STATA 10. 

## 3. Results

In total, 376 (82.8%) GPs returned the questionnaire and 374 had given information about open access. Among these, 31 (8.3%) GPs had walk-in open access every weekday and 22 (5.9%) had open access less often (mostly once a week). The characteristics of the study population are shown in Table 1. We found that 89 (24.1%, 95% CI: 19.8–28.8) of the GPs suffered from burnout.

Among GPs with open access, 13 (43.3%) suffered from burnout compared with 76 (22.6%) among GPs without open access. The crude OR for burnout was 2.9 (95% CI: 1.3–6.5, *P* = 0.009) for GPs with open access (Table 2). The adjusted analysis showed that GPs with open access had a 3.1 (95% CI: 1.1–8.8, *P* = 0.035) increased likelihood of suffering from burnout compared with GPs without open access.

We saw an association, although statistically insignificant, between walk-in open access and consultation time of 12 minutes or less (OR = 3.1, 95% CI: 0.9–10.1) and working more than 52 hours per week (OR = 3.1, 95% CI: 0.9–10.6). GPs with open access had a high likelihood of not participating in CME-group activities (OR = 4.0, 95% CI: 1.4–10.8).

## 4. Discussion

### 4.1. Main Findings

We found an association between being a GP in a practice with daily walk-in open access and suffering from burnout compared with GPs who did not provide open access on a daily basis. We also found that open access was associated with providing shorter consultations and working more than 52 hours per week. However, the cross-sectional nature of our design does not allow us to make causal inferences, for example, whether burnout was actually caused by open access or if burnout forces some GPs to introduce open access as a solution. Furthermore, we cannot determine whether burnout or open access were indicating variables of a problem causing burnout. 

### 4.2. Strengths and Weaknesses

The strength of this study lies in its use of an international validated scale for measuring burnout, its high response rate, and its fine statistical precision. The validity of the burnout measure has been demonstrated in cross-sectional studies. However, if the measure is unstable and depends too much on, for example, the job stress on a particular day (e.g., an open access day), the measure may be inaccurate. However, the MBI-HSS has been shown to be highly associated with more stable measures, for example, mental health and job satisfaction [17–19]. It is a weakness that we lack a clinically derived cutoff for the Maslach. However, in this study, where we analyse the association between low and high scores, this possible weakness can be ignored.

Although the study enjoyed a high response rate, a small group of physicians did not respond, which may introduce an element of selection bias. It may be assumed that GPs experiencing burnout would have less energy and enthusiasm to fill out the questionnaire. Given a higher prevalence of burnout among nonresponders, we may therefore have underestimated the prevalence of burnout. However, we do not know whether there was a selection bias among GPs with open access, but if they were less inclined to participate, we may have underestimated the association between open access and burnout.

### 4.3. Comparison with Other Studies

The prevalence of burnout among GPs found in this study is supported by the findings of a Swiss study which showed that one-third of primary care physicians experienced burnout [18]. We have been unable to identify studies examining associations between burnout and open access among GPs. 

Only little research has assessed the benefits and unfavourable effects of open access. Studies from the US have suggested that open access reduced waits and workload, increased continuity, improved efficiency, and allowed more patients to see their physician [1, 3]. In contrast, another US study of open access in five primary care practices showed an initial, large reduction in waits which disappeared and the waits actually increased over two years of followup and no change in no-show rates was observed [20]. Another US study found no good evidence for or against open access [21]. Perhaps our findings reflect that open access initially is a fine solution, but in the long run, it produces loss of control and increased workload.

Some of the same results are seen in the UK where the National Primary Care Collaborative introduced the principles of “Advanced Access” in 2000. A study among 462 general practices in England showed a 50% reduction in wait, but that only two-thirds of practices managed to produce an improvement [4] and that some vulnerable patient groups may be disadvantaged by the appointment system [22]. A controlled before-and-after study from England (48 practices) found that the wait for an appointment was slightly shorter with advanced access, but no differences were observed in meeting the NHS standard, the number of appointments offered, and patients seen or in continuity of care [23].

How patients evaluate open access has been investigated among 1153 adults consulting GPs in six practices. Appointment wait was only important if the appointment was for a child or when attending for a new health problem. Others were willing to wait if this meant that they would see their own doctor (especially patients with a long-standing illness, women, and older patients) or if they were able to attend an appointment at their own choice of time (most important for employed responders) [24]. This was confirmed in the previously mentioned controlled before-and-after study comparing 10 821 (84%) patients' priorities and experiences [25]. The top priority for patients was to be seen on the day of choice rather than to be seen quickly. Further, this study showed that although open access patients obtained their current appointment sooner than those in controlled practices, they were less likely to have been able to book in advance and were not more satisfied with the appointment system [25].

In an international perspective, our results show that more research in the association between burnout and the way GPs organise their appointments is needed. A European study showed a high proportion of doctors with high burnout [26]. Thus, the need for a better knowledge of how to prevent burnout is needed.

## 5. Conclusion

We found that GPs providing walk-in open access on a daily basis had a significant higher likelihood of suffering from burnout than those without open access. Although we cannot make causal inference from our study, it seems most appropriate to be observant and cautious in implementing open access in general practice. However, we do not know whether open access is a risk factor for burnout or if burnout forces the GPs to implement open access in order to cope with a high demand. We saw that open access was associated with providing shorter consultations and working longer hours per week. Hence, it may be that GPs got “trapped” in providing many consultations and that providing open access necessitates followup, information, and evaluation.

## Figures and Tables

**Table 1:** Characteristics of the participating 376 GPs. Mean age was 51.8 (SD = 6.7), mean weekly hours of work were 44.2 (SD = 8.8), and mean years in profession were 14.9 (SD = 8.4).

	*N*	%
Gender		
Men	228	61.0
Women	146	39.0
Marital status		
Married	345	93.0
Single	26	7.0
Age		
<45	58	15.6
45–49	86	23.1
50–54	81	21.7
55–59	102	27.4
>59	46	12.3
Practice organisation		
Solo practice	92	25.3
Group practice	32	8.8
Shared practice	93	25.6
Partnership practice	146	40.2
Open access (at least 30 minutes)		
Yes, every day	31	8.3
Yes, but less than every day	22	5.9
No	321	85.8
Number of patients per GP		
<1385	96	25.9
1385–1785	181	48.8
>1785	94	25.3
Number of consultations per GP in 2003		
<7720	96	25.9
7720–10.770	183	49.3
>10770	92	24.8
CME group		
Member of a CME and a supervision group	70	18.7
Being a member of either a CME or supervision group	274	73.3
Neither a member of a CME nor of a supervision group	30	8.0
CME days spent in 2 years on GP-relevant activities		
0–5 days	34	9.3
6–9 days	60	16.4
10–17 days	190	51.8
>17 days	83	22.6
Job satisfaction (Warr-Cook-Wall)		
>6.0	77	20.5
5.6–6.0	112	29.8
5.1–5.5	101	26.9
1.0–5.0	86	22.9
Burnout (Maslach score)		
No	280	75.9
Yes (EE > 26 and/or DP > 9)	89	24.1
Weekly working hours		
<37	52	14.7
37–41	90	25.4
42–46	95	26.8
47–51	66	18.6
>51	52	14.7
Minutes typically used per consultation		
≥13 min	294	78.8
<13 min	79	21.2

**Table 2:** Association between walk-in open access and burnout, consultation time, working hours, job satisfaction, and participation in CME activities.

	*N*	Open access (%)	Univariate			Multivariate		
			OR	95% CI	*P* value	OR^a^	95% CI	*P* value
Burnout (Maslach score)								
No	240	5.9	1	(Ref)		1	(Ref)	
Yes (EE > 26 and/or DP > 9)	66	15.4	2.9	1.3–6.5	0.009	3.1	1.1–8.8	0.035
Minutes typically used per consultation								
≥13 min	253	6.0	1	(Ref)		1	(Ref)	
<13 min	58	17.1	3.3	1.5–7.3	0.004	3.1	0.9–10.1	0.066
Weekly working hours								
<37	43	8.5	1	(Ref)		1	(Ref)	
37–41	83	5.7	0.6	0.2–2.5	0.533	0.7	0.2–2.8	0.662
42–46	86	7.5	0.9	0.2–3.2	0.838	1.2	0.3–4.1	0.793
47–51	57	8.1	0.9	0.2–3.7	0.933	1.2	0.3–4.6	0.747
>51	42	14.3	1.8	0.5–6.6	0.379	3.1	0.9–10.6	0.077
CME group								
Member of both CME and supervision group	59	9.2	1.4	0.5–3.6	0.521	1.5	0.4–5.3	0.539
Member of either CME or supervision group	230	6.9	1	(Ref)		1	(Ref)	
Neither member of CME nor supervision group	22	18.5	3.1	1.0–9.1	0.043	4.0	1.4–10.8	0.007
CME days spent in 2 years on GP-relevant activities								
0–5 days	55	3.5	4.4	0.8–25.6	0.099	8.2	1.6–43.1	0.013
6–9 days	25	13.8	1	(Ref)		1	(Ref)	
10–17 days	161	9.0	2.7	0.6–12.3	0.189	4.1	1.1–15.1	0.032
>17 days	67	6.9	2.1	0.4–11.0	0.401	2.9	0.6–13.2	0.177
Job satisfaction (Warr-Cook-Wall)								
>6.0	60	11.8	1	(Ref)		1	(Ref)	
5.6–6.0	98	3.0	0.2	0.1–0.9	0.035	0.2	0.0–1.1	0.071
5.1–5.5	84	10.6	0.9	0.3–2.4	0.822	0.7	0.2–2.7	0.648
1.0–5.0	69	9.2	0.8	0.3–2.2	0.617	0.5	0.1–1.8	0.301

OR^a^: adjusted odds ratio including sex, age, practice organisation, number of enrolled patients per GP, weekly working hours, number of consultations provided per GP, and participation in CME, corrected for clusters of GPs within the same practice.
